# Mesenchymal Stem Cell Protects Injured Renal Tubular Epithelial Cells by Regulating mTOR-Mediated Th17/Treg Axis

**DOI:** 10.3389/fimmu.2021.684197

**Published:** 2021-05-28

**Authors:** Yongsheng Luo, Jingjing Guo, Pingbao Zhang, Yin Celeste Cheuk, Yamei Jiang, Jiyan Wang, Shihao Xu, Ruiming Rong

**Affiliations:** ^1^ Department of Urology, Zhongshan Hospital, Fudan University, Shanghai, China; ^2^ Shanghai Key Laboratory of Organ Transplantation, Shanghai, China; ^3^ Shanghai Medical College, Fudan University, Shanghai, China

**Keywords:** mesenchymal stem cell, kidney injury, regulatory T cell, T helper 17 cell, mTOR

## Abstract

The increase in T helper 17 cell (Th17)-mediated pro-inflammatory response and decrease in regulatory T cell (Treg)-mediated anti-inflammatory effect aggravate renal tubular epithelial cell (RTEC) injury. However, increasing evidence indicated that mesenchymal stem cell (MSC) possessed the ability to control the imbalance between Th17 and Treg. Given that Th17 and Treg are derived from a common CD4^+^ T cell precursor, we summarize the current knowledge of MSC-mediated inhibition of the mammalian target of rapamycin (mTOR), which is a master regulator of CD4^+^ T cell polarization. During CD4^+^ T cell differentiation, mTOR signaling mediates Th17 and Treg differentiation *via* hypoxia-inducible factor-1α (HIF-1α)-dependent metabolic regulation and signaling pathway, as well as mTOR-mediated phosphorylation of signal transducer and activator of transcription (STAT) 3 and 5. Through interfering with mTOR signaling, MSC restrains CD4^+^ T cell differentiation into Th17, but in turn promotes Treg generation. Thus, this review indicates that MSC-mediated Th17-to-Treg polarization is expected to act as new immunotherapy for kidney injury.

## Introduction

Renal tubular epithelial cell (RTEC) injury due to ischemia–reperfusion injury (IRI), nephrotoxicity and other causes lead to a rapid decline in renal function and over-activated inflammatory immune response. If the inflammatory damage to RTEC was not controlled timely, a continuous renal injury would result in renal fibrosis and failure (1–3). Unfortunately, current pharmacological interventions have failed to prevent intrarenal inflammatory cascade. However, increasing studies showed that a diverse mesenchymal stem cell (MSC) population could form a balanced inflammatory microenvironment and protect injured RTEC by regulating mammalian target of rapamycin (mTOR)-mediated balance between pro-inflammatory T helper 17 cell (Th17) and anti-inflammatory regulatory T cell (Treg) (4–7).

In this review, we will introduce the imbalance between Th17 and Treg in RTEC injury, subsequently discuss MSC control for Th17 and Treg balance, and ultimately integrate current major mechanisms of MSC regulation on mTOR-mediated Th17 and Treg differentiation.

## The Imbalance and Functions of Th17 and Treg in RTEC Injury

Th17 characterized by the expression of specific transcription factor RAR-related receptor γ thymus isoform (RORγt) plays a vital role in RTEC damage by producing IL-17 and other pro-inflammatory cytokines (8, 9). However, through the specific immunoregulatory functions, Treg that express the transcription factor forkhead box protein P3 (Foxp3) can inhibit Th17-mediated inflammatory response, promote inhibitory cytokine secretion, and ultimately protect renal injury (5, 6, 10).

### Th17-Dominant Kidney Damage

Numerous studies indicated that Th17 participated as the main kidney infiltrating inflammatory mediator in RTEC injury due to ureteral obstruction, acute kidney injury, chronic kidney disease, etc (11–14). Moreover, some studies reported that the Th17 number and Th17/Treg ratio were positively associated with the level of renal injury. Meanwhile, Th17 deficient mice due to IL-17 or RORγt null significantly alleviated RTEC injury (15).

The predominant Th17 exerted a robust pro-inflammatory response in renal injury *via* the expressions of IL-17 and C−C−motif chemokine receptor (CCR) 6 (9, 16–18). For example, Kaneko et al. reported that Th17-secreting IL-17 bound to the corresponding receptor and promoted the expression of C-C motif chemokine ligand (CCL) 20, which attracted pro-inflammatory lymphocytes, dendritic cells, monocytes, and neutrophils to RTEC and ultimately led to immune-mediated damage to the kidney (9). Moreover, IL−17 was also found to increase CCL2 and IL−8 expressions in human proximal RTEC (19). Meanwhile, in obstructive renal injury, Th17 may contribute to the fibrotic transition from acute kidney injury to chronic kidney disease due to IL-17A expression (11, 12). In addition, some studies indicated that Th17 exerted distinct effector functions by necessarily migrating to target organs, which was mediated by chemokines [such as CCL20] and corresponding receptors [such as CCR6] (16, 19, 20). Moreover, Th17 was reported to induce CCL20 expression in RTEC, promoting the recruitment of other leukocytes to the kidney (19).

### Treg-Weak Renoprotection

A recent study of single-cell RNA-seq of renal immune cells showed that Treg in regenerating renal existed the high expression of tissue repair- and pro-angiogenesis-related genes. This finding suggested that Treg provided potential kidney protection from kidney damage (21). Indeed, Treg was reported to exert a key role in preventing kidney injury and facilitating renal repair (22–24). For instance, Treg can prevent inflammatory cell accumulation and promote anti-inflammatory M2-macrophage generation, which exerted a renoprotective function during acute or chronic renal injury (5, 10). In addition, some studies reported that Treg depletion in mice enhanced inflammatory response and aggravated kidney injury (25–28). Moreover, the deficiency of Foxp3 in *Rag1^-/-^* mice also caused severe kidney damage (28). These studies indicated Treg renoprotection.

Unfortunately, Cao et al. reported that low percentages of Treg in intrarenal leucocytes existed in mice renal at 24 h after IRI (25). Moreover, Dong et al. also found decreased Treg number in patients with acute kidney injury (29). Meanwhile, increasing studies indicated that lower Treg infiltration, compared with Th17, existed in the injured kidney (30–32). Some studies also found that the adoptive transfer of Treg reduced pro-inflammatory IFN-γ and TNF-α generation, improved kidney function, and relieved acute tubular necrosis after migration to the postischemic kidney (33–35). These transferred cells were also able to inhibit innate immune-related renal injury *via* switching ATP into adenosine mediated by CD73 on the surface of Treg. Subsequently, the adenosine bound to the A_2a_ receptor and then enhanced the expression of PD-1, which exerted a vital renoprotective effect during IRI (34, 36).

## The Balance Between Th17 and Treg Controlled by MSC

MSC is a fibroblast-like cell population extracted from fat, bone marrow, umbilical cord, and other tissues, with an immune privilege due to low immunogenicity (8). Recent studies showed that the most intriguing role of MSC was the immunomodulatory effect on the CD4^+^ T cell polarization, with induction of Treg and suppression of Th17 differentiation (9, 37). For example, in an arthritis model, human MSC infusion could reduce Th17 number, promote Treg generation and enhance IL-10 production (38). Ghannam et al. reported that MSC induced a regulatory Th17 generation with RORγt downregulation and FOXP3 upregulation, which suppressed T cell proliferation and alleviated inflammation (39). Therefore, MSC-mediated Th17-to-Treg polarization creates anti-inflammatory processes and then protects injured RTEC.

### The Enhancement of Treg Effect

Hu et al. reported that MSC infusion increased Treg proportion in the renal and spleen, protecting the injured kidney (40, 41). Moreover, Casiraghi et al. found that transfer of MSC expanded Treg proportion in lymphoid organs and further prolonged kidney allograft survival (42). Similarly, autologous transfer of MSC to patients after kidney transplantation possessed the ability to generate Treg (43). Indeed, it was demonstrated that Treg depletion eliminated MSC protection for organ injury (44).

It is well established that Treg and Th17 derive from a common CD4^+^ T cell precursor, which offers the potential for MSC-mediated CD4^+^ T cells differentiating into Treg but not Th17 (45). For instance, English et al. reported that CD4^+^ T cells and MSC coculture suppressed lymphocyte proliferation due to an increased Treg differentiation (46). Meanwhile, Liu et al. reported that the coculture of T cells and mice MSC showed a significant increase in Foxp3 expression and Treg proportion (47), which was similar to the human MSC coculture experiment (48). Moreover, both *in vivo* and *in vitro*, MSC favored Treg generation and their immunosuppressive function (49).

### The Attenuation of Th17 Effect

Numerous studies indicated that the inhibition of Th17 and corresponding function could act as effective methods of protecting renal injury (12, 50–52). In vitro experiments from rat and human indicated that MSC possessed the ability to decrease Th17 generation from naive T cells and inhibit IL-17 and IL-22 secretion from Th17 (39, 53). Meanwhile, *in vivo*, the application of MSC suppressed EAE progress by reducing the secretion of IL-17 and IL-23 (54).

Furthermore, Th17 was reported to possess the ability to transdifferentiate into Foxp3^-^IL−10^+^ type 1 Treg (Tr1) with suppressive properties (55, 56). Moreover, MSC also was found to promote Foxp3 expression with increased IL-10 secretion, but suppress RORC expression with reduced IL-17 and IL-22 in differentiated Th17. Meanwhile, MSC stimulated by inflammatory cytokine showed a high CD54 expression, which contributed to the adhesion of Th17 to MSC and the induction of T cell with regulatory features (39). These findings suggest a new approach of treating injured RTEC by intervening pathogenic Th17 into suppressive Th17.

In addition, some studies indicated that Treg also possessed the ability to inhibit Th17-mediated kidney injury (12, 45, 57, 58). Particularly, recent studies reported a specialized Treg type 17 that colocalized with Th17 and exclusively inhibited Th17-mediated effect *via* spatial interaction, IL-10 production, and CCR6 expression (19, 59).

## MSC-Mediated mTOR Inhibition Regulating Th17/Treg Axis

**Figure 1:**
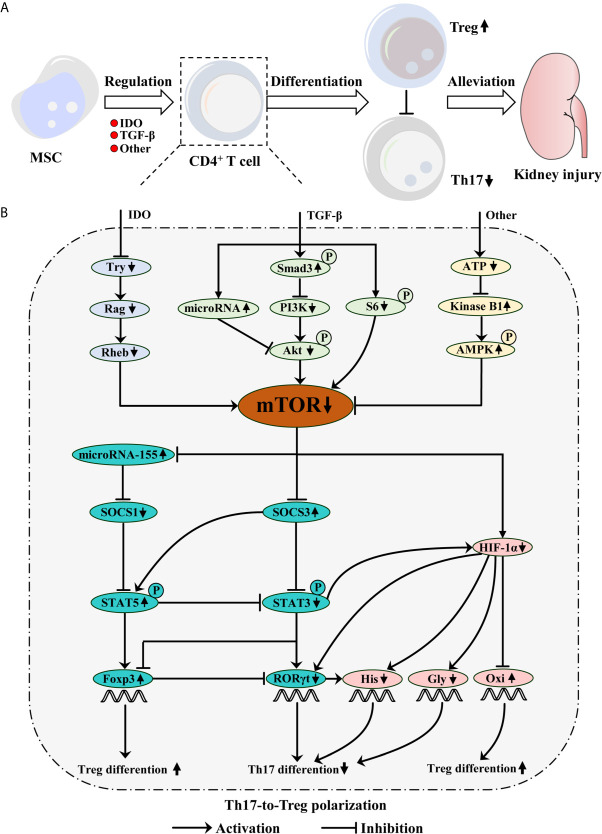
Mesenchymal stem cell (MSC) protects kidney injury *via* mTOR-mediated Th17-to-Treg polarization. **(A)** MSC inhibits mTOR signaling during CD4^+^ T cell differentiation *via* IDO and TGF-β secretions as well as other mechanisms, which restrains Th17 differentiation, but promotes Treg generation. Consequently, MSC-mediated Th17-to-Treg polarization alleviates kidney injury. **(B)** TGF-β phosphorylates Smad3, which inhibits PI3K/Akt/mTOR pathway. TGF-β also inhibits S6-mediated mTOR signaling. Moreover, TGF-β promotes microRNA expression, which can inhibit Akt/mTOR pathway. IDO can deplete tryptophan (Try), which results in mTOR inhibition mediated by the Rag and following Rheb. Additionally, MSC-mediated low ATP concentration increases kinase B1, subsequently phosphorylates AMPK, ultimately inhibits mTOR signaling. However, mTOR signaling can inhibit microRNA-155 and SOCS3 expression, but promote HIF-1α generation. Briefly, microRNA-155 inhibits SOCS1 expression, which can restrain STAT5 phosphorylation. The phosphorylation of STAT5 not only restrains STAT3 phosphorylation, but also upregulates Foxp3 expression, which promotes Treg differentiation. SOCS3 can promote STAT5 phosphorylation, but inhibit STAT3-mediated HIF-1α and RORγt expression, which ultimately reduces Th17 differentiation. Moreover, STAT3 possesses the ability to inhibit Foxp3 expression. HIF-1α can promote RORγt expression and increase histone acetylation (His) with RORγt collaboration, which possesses the ability to induce Th17 differentiation. Meanwhile, HIF-1α inhibition can promote Treg but not Th17 differentiation by mediating glycolysis (Gly) switching into the oxidative phosphorylation (Oxi).

Mechanistically, MSC regulates the balance between Th17 and Treg predominantly *via* indoleamine 2, 3-dioxgenase (IDO) and TGF-β secretions as well as other mechanisms, which mimic the rapamycin effect of inhibiting mTOR, including mTOR complex 1 (mTORC1) and 2 (mTORC2) (8, 9, 60–65). Accumulating evidence indicates that mTOR, a serine/threonine kinase, acts as a key regulator of metabolic programmers and signaling pathways in CD4^+^ T cell differentiation (62, 66, 67). For example, stimulating mTOR activation in CD4^+^ T cell drove glycolytic metabolism and promoted RORγt expression. However, MSC-mediated mTOR downregulation during CD4^+^ T cell differentiation induced Treg generation by fostering oxidative metabolism and Foxp3 transcription. Thus, MSC-mediated mTOR inhibition in CD4^+^ T cell promotes Th17-to-Treg polarization, conducive to protecting injured RTEC (5, 6, 8, 62–64) (**Figure 1**).

### MSC Inhibition on mTOR Signaling

Several studies showed that MSC-producing IDO could inhibit Th17 differentiation, induce Treg generation, and subsequently prevent renal injury (9, 61, 64). IDO is the main enzyme for promoting tryptophan catabolism into kynurenine. However, tryptophan was reported to possess the ability to activate the Rag complex, which recruited and linked mTORC1 to Rheb on the lysosomes. Subsequently, mTORC1 was activated due to the spatial regulation of Rheb and Rag (68–70). These findings may explain the phenomenon that IDO-mediated tryptophan exhaustion caused mTORC1 (as a nutrient sensor) inhibition, which further suppressed Th17 number and function but promoted Treg generation. Moreover, IDO depletion or tryptophan supplement reversed the effects (9, 61, 62, 64).

In addition to IDO, TGF-β secreted by MSC plays a role for inducing Treg generation due to mTOR inhibition (8, 61, 63). Priyadharshini et al. found that exposure of Treg to TGF-β repressed S6 and Akt phosphorylation targeting mTORC1 and mTORC2, respectively. Moreover, TGF-β reprogrammed Treg metabolism by inhibiting PI3K-mediated mTOR signaling (63). These results are in line with the report that Smad3 phosphorylation mediated by the interaction between TGF-β/TGF-β receptor limited CD4^+^ T cell proliferation and inhibited classic PI3K/Akt/mTOR pathway, which resembled rapamycin inhibition of mTOR signaling (65). Moreover, *in vitro*, the stimulation of TGF-β altogether with all-trans retinoic acid promoted the expression of a set of microRNAs such as microRNA-15b/16, which inhibited Akt/mTOR signaling and further induced Treg generation (71, 72).

Additionally, Yoo et al. demonstrated that MSC suppressed CD25 expression on the surface of T cells *via* increased liver kinase B1 and AMP-activated protein kinase phosphorylation induced by low adenosine triphosphate (ATP) concentration (66). Liver kinase B1, a serine/threonine kinase, can increase phosphorylation of AMP-activated protein kinase and then inhibit mTORC1 signaling, which ultimately reduces inflammatory cytokine secretion in T cells (60, 66).

### mTOR-Mediated Th17/Treg Axis

Increasing studies showed that mTOR-low populations of CD4^+^ T cells with increased oxidative phosphorylation were likely to become Treg with high signal transducer and activator of transcription (STAT) 5 and Foxp3 expression, while mTOR-high populations with enhanced hypoxia-inducible factor-1α (HIF-1α)-mediated glycolysis were enriched for Th17 with high STAT3 and RORγt expression (22, 73, 74). Moreover, the transfer of Treg pretreated by pharmacological mTOR inhibition to kidney injury mice following by IRI can improve the renal function recovery and reduce kidney fibrosis due to enhanced immunoregulatory effect of Treg (75). These studies indicated that mTOR inhibition played a vital role in renal protection by promoting Treg but not Th17 differentiation, which involved mTOR-dependent energy metabolism and protein translation networks.

#### mTOR/HIF-1α-Mediated Metabolic Reprogramming

With CD4^+^ T cell activation, some glycolytic molecules, such as glyceraldehyde-3-phosphate dehydrogenase, lactate dehydrogenase and glucose transporters were upregulated to promote glucose uptake due to the increased bioenergetic demands (66). The process can be regulated by mTOR-mediated signaling, such as the transcription factor HIF-1α expression (76). For example, mTOR was reported to promote STAT3 activation, which increased HIF-1α expression *via* direct binding of STAT3 to the HIF-1α gene promoter region (45, 62). Subsequently, increased HIF-1α can mediate oxidative phosphorylation switching into aerobic glycolysis by targeting glycolytic genes such as glycolytic enzymes hexokinase 2, monocarboxylic acid transporter member 4, and glucose transporter 1, which are vital for Th17 differentiation and IL-17 expression (22, 62). Consequently, HIF-1α-mediated glycolytic molecules act as main metabolic checkpoints to regulate CD4^+^ T cell polarization. For instance, HIF-1α deficiency in CD4^+^ T cells resulted in less Th17 and IL-17 generations by diminishing glycolytic molecule expression (22, 45, 62). In addition, the glucose analogue 2-deoxyglucose was also reported to promote Treg differentiation but dampen the generation of Th17 during CD4^+^ T cell polarization by inhibiting key glycolytic molecules (22). Therefore, HIF-1α-dependent metabolic reprogramming mediated by mTOR signaling distinguishes the lineage decisions between Treg and Th17.

#### mTOR/HIF-1α-Mediated Signaling Pathway

In addition to metabolic regulation, mTOR-mediated HIF-1α also directly binds to RORγt gene promoter region and subsequently promotes RORγt transcription. Moreover, HIF-1α with RORγt collaboration activates p300-mediated histone acetyltransferase, which increases histone acetylation, opens up the chromatin structure, and ultimately facilitates Th17 differentiation (45, 77). Additionally, HIF-1α was reported to activate the process that promoted the degradation of Foxp3, which inhibited the differentiation of Treg. Foxp3 degradation may explain that Foxp3^+^RORγt^+^ Treg/TH17 precursors committed to the differentiation of Th17 by diminishing Foxp3 transcription (45). Moreover, HIF-1α could drive the IL-23 receptor upregulation, vital for IL-17 and Th17 generation (22, 78). These results were in line with the report that HIF-1α has high expression during the differentiation of Th17, while Treg showed a low level of HIF-1α (26). Correspondingly, HIF-1α absence mediated by mTOR inhibition impaired Th17 differentiation as well as IL-17 and IL-23 receptor expressions, but upregulated Foxp3 expression (22, 79). Meanwhile, Foxp3 upregulation antagonizes RORγt expression and Th17 generation. Furthermore, Shi et al. also indicated that Ctla4 and Gpr83 molecules on the surface of Treg were upregulated due to HIF-1α absence (22). Therefore, mTOR-mediated HIF-1α signaling would be expected to act as a key regulator of CD4^+^ T cell polarization.

#### mTOR/STATs-Mediated Signaling Pathway

Several studies showed that mTORC1 could activate STAT3 by inhibiting the suppressor of cytokine signaling (SOCS) 3 expression, which could repress STAT3 phosphorylation or induce proteasomal degradation of STAT3 (76, 80, 81). Moreover, mTORC1 inhibited STAT5 activation by downregulating microRNA-155, an inhibitor of the SOCS1 (82–84). Similarly, mTORC1 inhibition was reported to increase the phosphorylation of STAT5 by inhibiting SOCS3 expression (76, 85).

Accumulating studies showed that STAT3 induced RORγt and IL-17 expressions, while the deficiency of STAT3 impaired the expressions of RORγt and IL-17, but increased Foxp3 expression (62, 76, 86, 87). However, through competition with STAT3, STAT5 could bind to IL-17a gene sites, subsequently modifying the IL-17 gene locus due to a decrease in histone-3 lysine-4 trimethylation (IL-17 expression promoter) and increase in histone 3 lysine 27 trimethylation (IL-17 expression inhibition) (62, 87). Moreover, STAT5 activation was reported to promote the differentiation of Treg by upregulating the expression of Foxp3 (76, 88, 89). Interestingly, several studies indicated that Foxp3^+^RORγt^+^ Treg/TH17 precursors under an anti-inflammatory environment (such as high TGF-β levels without IL-6) showed a high Foxp3 expression that antagonized the binding of RORγt to DNA *via* an exon 2-encoded sequence interaction, which inhibited IL-17 expression and further pushed T cell differentiation towards the Treg. Reversely, given that STAT3 activation stimulated by pro-inflammatory cytokines (such as IL-21 or IL-6 with low TGF-β levels) overcame Foxp3 antagonism of RORγt, the precursors tended to IL-23 receptor upregulation and subsequently pushed T cell differentiation into Th17 (45, 90). Therefore, the activation of STAT3 or STAT5 mediated Th17 or Treg differentiation.

## Conclusions and Perspectives

Currently, MSC has been extensively reported as a promising therapy for renal injury due to its renoprotection for injured RTEC (91–93). However, the therapeutic effect of MSC was greatly weakened in the light of the fact that a majority of MSC were stuck in the lungs after adoptive transfer, with a small part reaching to the spleen, liver, renal, and other organs (1, 94). In view of a similar mechanism of mTOR inhibition, the transfer of MSC combination with rapamycin was also reported to exert immunosuppressive effect and protect injured RTEC to a higher degree (66). Although MSC-mediated Th17-to-Treg polarization mainly depended on mTOR inhibition, the immunoregulatory mechanism was complex and not fully understood. Therefore, a successful application would rely on deep exploration and further resolution of the unanswered question.

## Author Contributions

YL, JG, and PZ conceived the conception, performed the literature search and wrote the manuscript. YC, YJ, JW, and SX performed the literature search and participated in the writing. RR conceived the conception and revised the manuscript. All authors contributed to the article and approved the submitted version.

## Funding

This work was supported by the National Key R&D Program of China (2018YFA0107501) and National Natural Science Foundation of China (81770747 and 81970646).

## Conflict of Interest

The authors declare that the research was conducted in the absence of any commercial or financial relationships that could be construed as a potential conflict of interest.
